# Local autoimmune encephalomyelitis model in a rat brain with precise control over lesion placement

**DOI:** 10.1371/journal.pone.0262677

**Published:** 2022-01-21

**Authors:** Lukasz Kalkowski, Dominika Golubczyk, Joanna Kwiatkowska, Malgorzata Domzalska, Piotr Walczak, Izabela Malysz-Cymborska

**Affiliations:** 1 Department of Neurosurgery, School of Medicine, Collegium Medicum, University of Warmia and Mazury, Olsztyn, Poland; 2 Center for Advanced Imaging Research and Department of Diagnostic Radiology and Nuclear Medicine, University of Maryland School of Medicine, Baltimore, MD, United States of America; Texas Biomedical Research Institute, UNITED STATES

## Abstract

Development of a novel, animal model for multiple sclerosis (MS) with reproducible and predictable lesion placement would enhance the discovery of effective treatments. Therefore, we would like to combine the advantages of the demyelination model with experimental autoimmune encephalomyelitis (EAE) to provide a local autoimmune encephalomyelitis (LAE) inside rat brain. We induced a demyelinating lesion by immunizing male Wistar rats, followed by blood-brain barrier opening protein (vascular endothelial growth factor) by stereotactic injection. We confirmed the immunization against myelin epitopes and minor neurological impairment. Histological assessment confirmed the lesion development after both 3- and 7 days post-injection. Our approach was sufficient to develop a demyelinating lesion with high reproducibility and low morbidity.

## Introduction

Multiple sclerosis (MS) is an autoimmune demyelinating disease and is one of the most widespread neurological conditions, with more than 2 million people suffering worldwide [1]. Even though several therapeutics show efficacy in treatment of MS [2, 3], none of them are capable of curing the disease. This ineffectiveness is due in part to insufficient understanding of the pathomechanism of autoimmunity in the central nervous system (CNS) in MS patients. Moreover, the unpredictable and variable disease course can be misleading in concluding the positive effect of therapies. Therefore, there is an urgent need to study the disease and test therapies in animal models. It is crucial to develop animal models that reflect the clinical complexity of the disease yet provide reproducibility of result and tools to reliably assess efficacy of tested therapies.

So far, the “gold standard” in preclinical MS research is an experimental autoimmune encephalomyelitis (EAE) model, developed mostly in mice and rats. Inducible pathology of this model includes MS-like lesions throughout CNS which is developed by immunization against myelin epitopes. The model, developed nearly a century ago [4], is well-known and was crucial in development of most drugs used to treat MS today [5]. However, EAE model has some significant disadvantages. Disseminated and randomly distributed demyelinating lesions complicate testing utility of novel therapies, such as cell transplantation or targeted delivery of therapeutics in particular. Moreover, the autoimmune demyelinating lesions are placed randomly, mostly in brainstem and spinal cord, in contrary to clinical MS, which mostly affects cerebral areas. In addition, disease severity in classic EAE varies depending on animal strain, but the mortality rate is frequently relatively high [6]. The field of MS research would greatly benefit from overcoming these disadvantages of animal models, enabling more accurate testing of potential therapeutic strategies. Therefore, we report on a new model of local autoimmune encephalomyelitis (LAE), that allows for precise and reproducible placement of demyelinating lesion on demand within desired brain structure. Our approach is based on inducing systemic, subclinical (without any noticeable neurological effect) immunization against brain epitopes with the mixture of bovine spinal cord tissue homogenate and complete Freund’s adjuvant in rats. Later, lesion formation is triggered by stereotactic injection of vascular endothelial growth factor (VEGF) which causes local opening of the blood-brain barrier (BBB). The resulting autoimmune demyelinating lesion is spatially limited to the proximity of VEGF injection, with typical MS-like perivascular cuffs, and demyelinating area with leukocyte infiltrates. This approach enables highly reproducible MS model that is ideally suited for rigorous assessment of therapeutic efficacy of novel drugs.

## Materials and methods

### Animals

All procedures were approved by Local Ethics Committee in Olsztyn (approval 59/2019) and were performed in accordance with the ARRIVE guidelines. Male, 7–8 weeks old Wistar rats (n = 25, 220–250 g body weight) were used for the experiments. Animals were kept on a 12/12 h day and night cycle and housed individually in cages, having at least 250 cm^2^ of free space each, with additional environmental enrichment (for example, nesting material), with free access to food and water. In order to minimize animal stress and suffering, animals were acclimated for further procedures (handling, using anesthetic chamber, weighting, performing cylinder test).

Animals were randomly divided into five groups (n = 5 each) and treated as listed in **Table 1**. Moreover, animals were observed and weighted daily from the day 0 (immunization day) to assess the effect of the procedures on general well-being of the animals.

**Table 1:** Overall experimental design with specific timepoints.

Treatment	LAE		Immunization only	Sham-operated	
	3 days survival	7 days survival		VEGF injection	PBS injection
Immunization	Day 0	Day 0	Day 0	-	-
VEGF injection	Day 14	Day 14	-	Day 14	Day 14
Euthanasia	Day 17	-	-	-	-
	-	Day 21	Day 21	Day 21	Day 21

### Immunization protocol

Bovine spinal cord homogenate was used as an immunogenic compound. Spinal cord tissue samples were collected from local slaughterhouse. Next, the samples were washed from blood clots and dura mater. Tissue was homogenized (1 g per 2 ml of ice-cold phosphate-buffered saline; PBS) using MagNA Lyser (Roche). Later, the volume corresponding to 200 μg of tissue was emulsified in complete Freund’s adjuvant (Rockland) in 1:1 v/v, giving a final dose of approx. 500 μl. For immunization, the animals were anesthetized with 3% isoflurane in oxygen and subdermal injection of immunogen was performed bilaterally, in tail base, at day 0.

### Intracerebral injection

On day 14, animals were anesthetized with 3% isoflurane and butorphanol (0.4 mg/kg i. m.) and placed in stereotactic frame (Kopf Instruments). Skin incision and a burr hole in skull was made. The coordinates from bregma were set at: -2.1 a/p, +3.2 mm m/l., -6.5 mm d/v. Next, the 10 μl (1μg) of VEGF (VEGF_164_, R&D Systems) solution in PBS was injected (0.5 μl/min) into right internal capsule using 33G Hamilton syringe and needle with micromanipulator (Kopf). To avoid outflows, needle was kept for 5 min post-injection. Later, the needle was removed, skin was sutured, and animals were awakened from anesthesia. Corresponding sham-operated group received an equal volume of PBS. For 2 days after surgery, animals received injections of analgesic drug (ketoprofen 5 mg/kg, i. m.).

### Behavioral assessment

Behavioral assessment with cylinder test was carried out in a blinded manner, 3 days before stereotactic injection and at the day of euthanasia. Animals were placed in a glass, transparent cylinder (190 mm in diameter, 225 mm height) for 2 min and the video was recorded. A set of mirrors was placed behind the cylinder to obtain a 360-degree observation inside the cylinder. Video recordings were analyzed using a slow-motion video recorder to determine the number of contacts by both forelimbs and by either impaired or non-impaired forelimbs. Results was presented as a fraction of total use of left and right forelimb and both simultaneously [7].

### Serum antibodies tittering

Blood samples were collected from portal vein into Vacumed® clot activator tubes (FL Medical) just before euthanasia. Serum was obtained and stored in -80˚C for anti-bovine spinal cord homogenate and anti-rat recombinant myelin oligodendrocyte glycoprotein (rrMOG) antibodies tittering, modified from All et al. [8]. Ninety-six-well plates were coated overnight with bovine spinal cord homogenate solution (50 μg/ml) or 1 μg/μl rrMOG_1-125_ peptide, respectively. Next, 100 μl of blocking buffer, composed from 2% bovine serum albumin (BSA; Sigma), in 0.01 M PBS with 0.1% Tween 20 (Sigma) was added to each well and incubated for 1.5 h at room temperature (RT), after which, solution was removed. Serial dilutions of rat serum (50 μl, starting from 1:100 diluted in blocking buffer), non-diluted and blank sample was added (each sample in duplicate) and incubated for 2 h at RT. Next, solution was removed, plate was washed with PBS with 0.1% Tween 20 and incubated for 1 h in RT with 50 μl of biotin-labeled anti-rat antibody (1:2000 in blocking buffer; Vector Labs). After incubation and washing with PBS, streptavidin-horseradish peroxidase (HRP) complex (1:2000 in blocking buffer; Vector Labs) was added and incubated in RT for 30 min. Solution was removed and after washing step, 3,3′,5,5′-Tetramethylbenzidine (TMB) substrate kit (Vector Labs) was used to visualize the immunoreactive complexes. The reaction was stopped by adding 50 μl of 1 M sulphuric acid into each well. Absorbance was measured at 450 nm (LEDetect, Labexim Products).

### Histology

Animals were anesthetized in 3% isoflurane and euthanized by intraperitoneal injection of sodium pentobarbital (150 mg/kg), then transcardially perfused with PBS-buffered 4% paraformaldehyde (PFA; Sigma). Brain tissue was harvested and post-fixed in 4% PFA for 24 h, cryopreserved in 30% sucrose and frozen on dry ice. Tissue samples were kept at -80°C for further analysis. Ten microns thick coronal cryosections were prepared. For histology, standard hematoxylin-eosin (H/E) and eriochrome cyanine R staining were performed as previously described [9, 10]. Immunofluorescent staining was performed using primary antibodies against: glial fibrillary acidic protein (GFAP; 1:500; Dako), rat IgG (1:500; Thermo Scientific), CD31 (1:50; Dako), CD45 (1:100; Dako), CD68 (1:100; Bio-Rad) and CD106 (1:50; Bio-Rad); with complementary Alexa Fluor® conjugated secondary antibodies (Thermo Scientific). Slides were mounted using FluoroShield® with 4′,6-diamidino-2-phenylindole (DAPI; Sigma).

### Image analysis

For H/E and eriochrome cyanine R staining, slides were processed with histological scanner, equipped with 40x objective (Panoramic Midi; 3DHistech). For immunofluorescent staining, images were taken using 20x and 40x objectives from inverted fluorescent microscope (Axio Observer; Zeiss). Additional downstream image processing was made using ImageJ software package (National Institutes of Health). Representative coronal sections from eriochrome stained tissue samples were used for measuring the area of demyelination. Images taken from IgG-stained tissue sections from ipsilateral hemisphere were analyzed. Ten circular regions of interest (ROIs; 400 μm diameter) were drawn to obtain a fluorescence intensity profile within the internal capsule and adjacent thalamic structures in coronal sections. Moreover, a DAPI-stained 8 ROIs (300 μm diameter) within the lesioned area were drawn to analyze the leukocyte infiltration degree. The data was counted as a total area of DAPI-positive signal within the ROIs, compared between the experimental and control groups and as a mean percentage of DAPI-positive signal area.

### Statistical analysis

The statistical analysis was made using Prism 8 software (GraphPad). A Shapiro-Wilk test was used to test the normality of the data. Kruskal-Wallis test was used to analyze the differences between the lesioned area in eriochrome stained tissue samples. One-way ANOVA with Tukey’s post-hoc test was performed to analyze the changes between the treatment groups and within the timepoints. A confidence value was set at 0.95. The numerical data was displayed as a mean with standard deviation.

## Results

### Systemic immunization procedure

**Fig 1:**
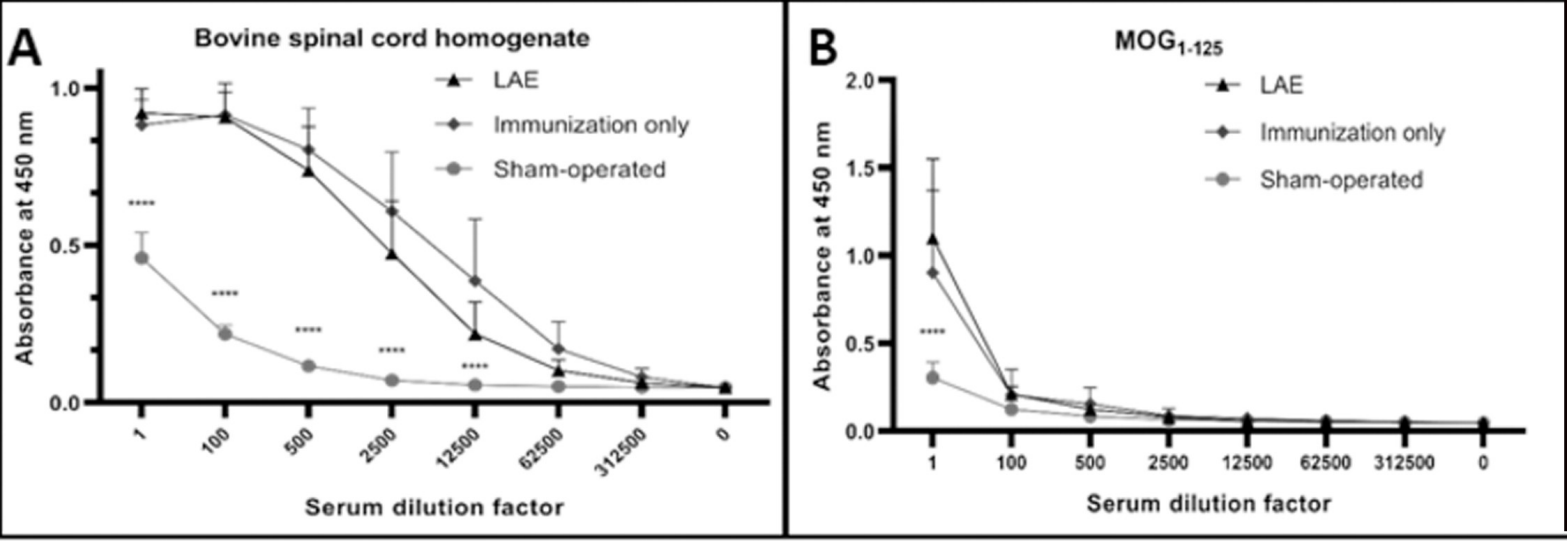
Antibody tittering against brain homogenate (A) and rrMOG_1-125_ (B) results, displayed as an absorbance for specific serum dilution factor. Results are displayed as a mean with standard deviation. P value of less than 0.0001 was presented as a “****”.

The immunization protocol was effective in sensitization to brain antigens. Serum antibody titer analysis, used for detection of antibodies against specific epitopes indicated that in all immunized groups, development of autoantibodies was robust (*p* < 0.0001) (**Fig 1**). This phenomenon was observed in case of, bovine spinal cord homogenate (**Fig 1A**), the immunogen, as well as MOG_1-125_ peptide (**Fig 1B**), confirming that the autoimmune response is robust against the myelin epitopes. The serum autoantibodies titer was similar between immunized groups, and significantly higher than in non-immunized animals. Thus, the autoimmunity is fully developed and is brain-specific, confirmed as detection of autoantibodies against MOG_1-125_.

### Disease course and behavioral assessment

**Fig 2:**
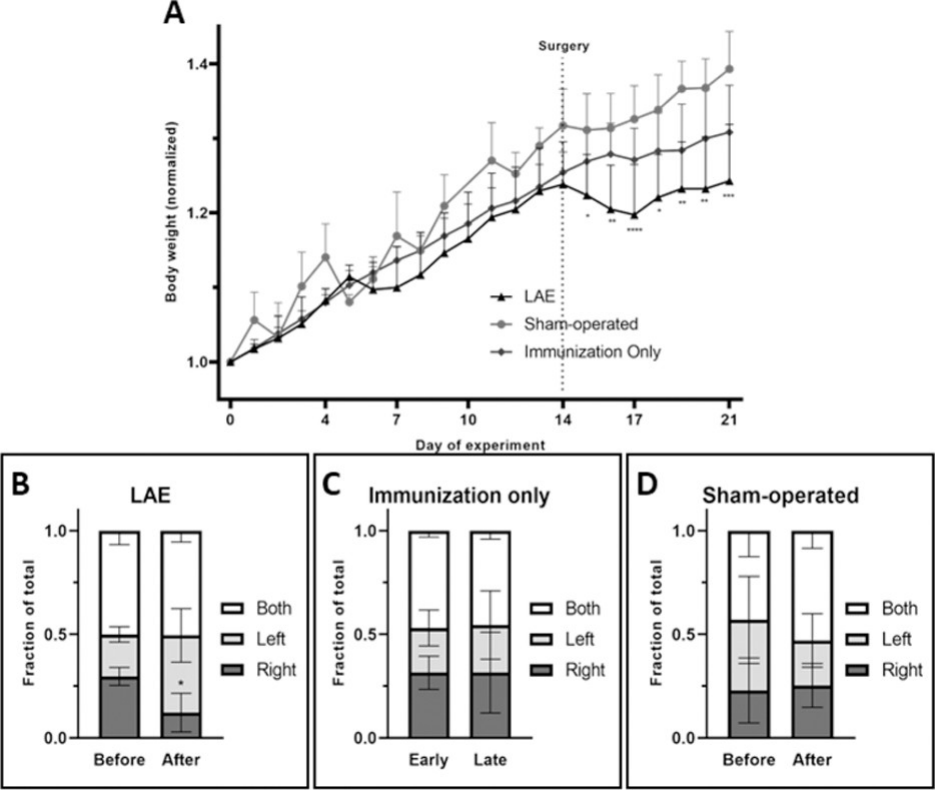
Animal body weight presented as a mean with standard deviation for specified treatment group (A) with behavioral assessment results (B-D). Changes in forelimb usage was presented as a fraction of total recorded touches for groups treated with full treatment (B), VEGF only (C) and with PBS only (D). P value of less than 0.05 was presented as “*”, <0.01 as a “**”, <0.001 as a “***” and <0.0001 as a “****”.

Daily bodyweight measurements were made for potential disease progression assessment. Animal body weight was normalized to day 0 to detect the possible changes independent of the initial body weight of the animals. To assess potential behavioral deficits following focal autoimmunity where unilateral lesion development is expected, we used the cylinder test. The results of both body weight measurements and behavioral testing indicate that immunization itself did not elicit EAE-specific weight loss as seen in the group with immunization only **(Fig 2A)**. Moreover, the surgery (stereotactic injection) resulted in only minimal weight loss that returns to baseline after 2 days. Compared to sham-operated animals, there was significant (*p* < 0.05) weight loss after VEGF stereotactic injection in LAE animals. Moreover, the behavioral assessment detected changes in forelimb usage symmetry (**Fig 2B–2D**). Comparing to control groups, the animals with LAE display the more pronounced use of the left forelimb during climbing through the cylinder wall (**Fig 2B**).

### Histological evaluation

**Fig 3:**
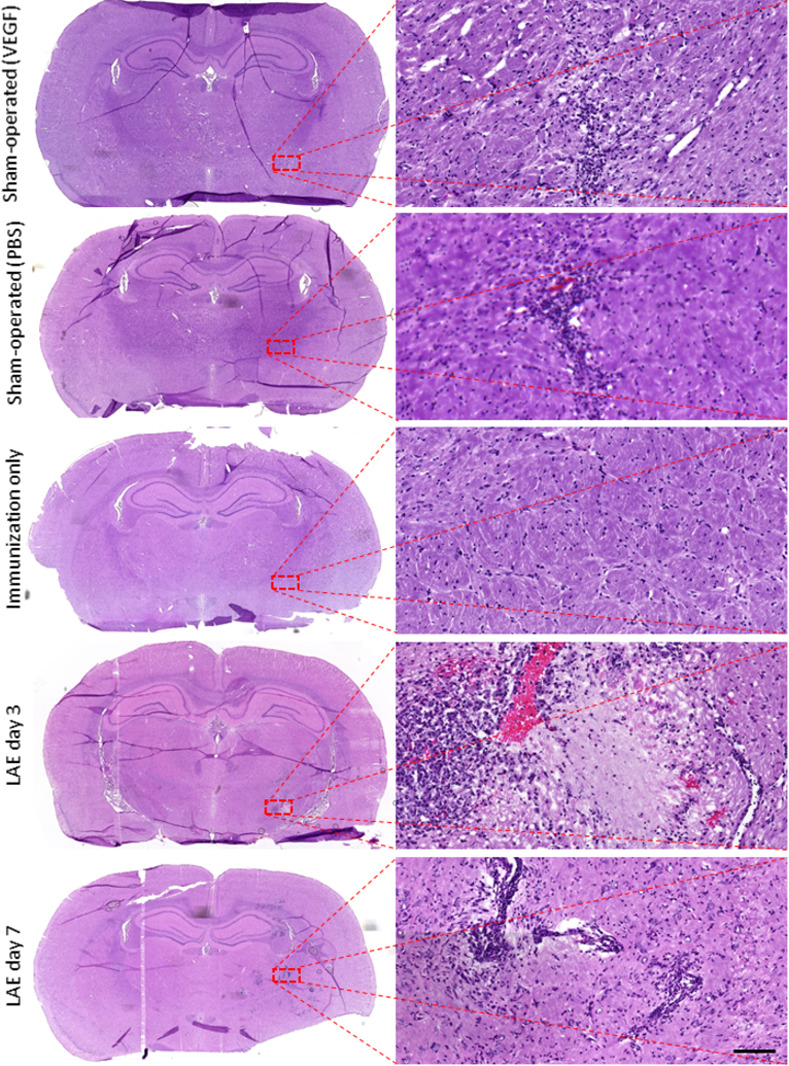
Hematoxylin/eosin stained brain tissue samples from all of the experimental groups. Higher magnification images are taken from the lesioned area (rectangle), scale bar represents 50 microns.

Histological staining (H/E) for sham-operated animals (**Fig 3**) showed only small region of hypercellularity at the site of needle insertion indicating mild microglial response. The injection area was without perivascular cuffs, infiltrating leukocytes or demyelination (**Fig 3**). Also, none of any pathological findings were found in group with immunization only (**Fig 3**). In animals with LAE (immunization+VEGF), quite extensive local perivascular cuffing was observed in the proximity of VEGF injection in the internal capsule, but also in surrounding brain area in H/E-stained tissue in both 3- and 7 days post-op (**Fig 3**). Moreover, more extensive leukocyte infiltrates were observed in the internal capsule in animals with LAE 7 days post-op (**Fig 3**).

**Fig 4:**
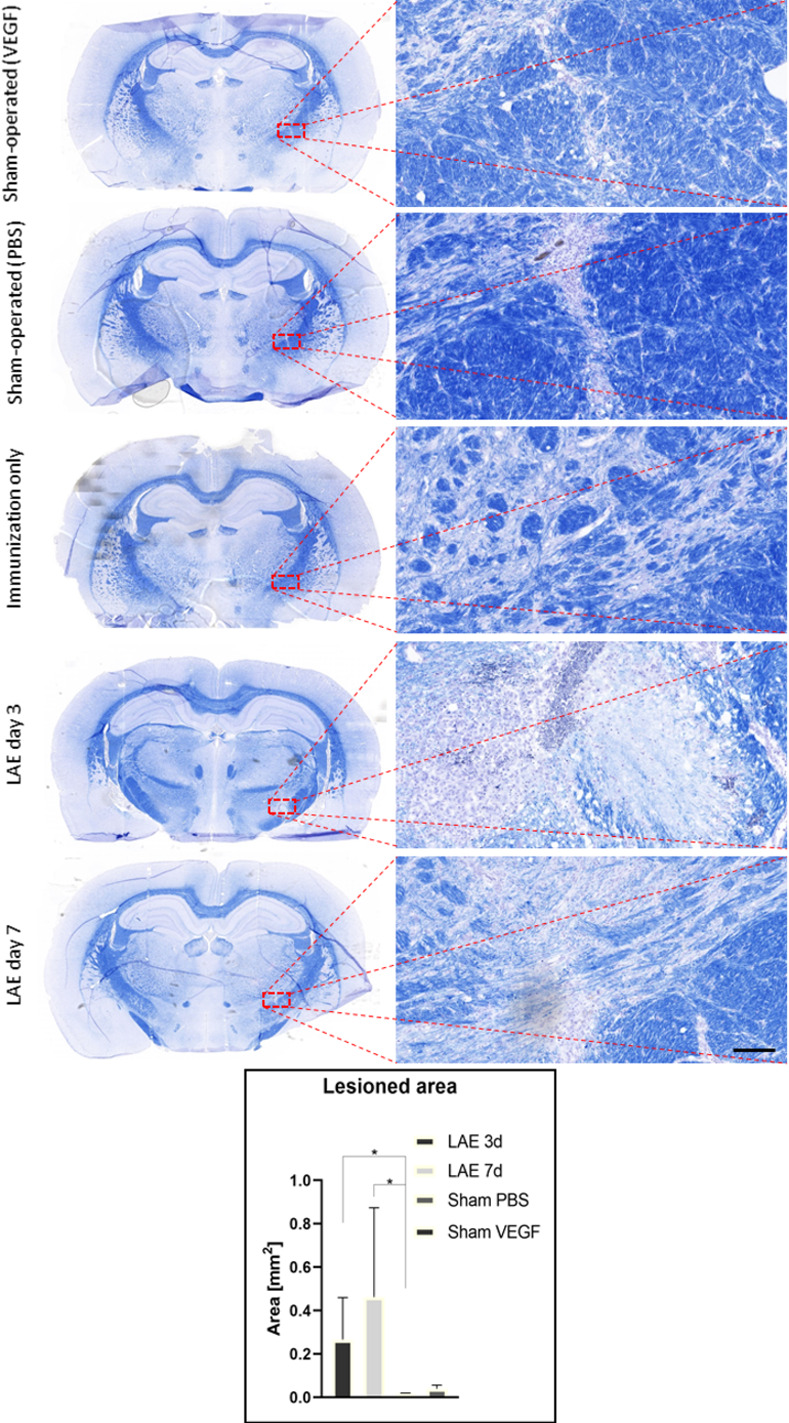
Eriochrome stained brain tissue samples from all of the experimental groups. Higher magnification images are taken from the lesioned area (rectangle), scale bar represents 50 microns. The graph illustrates the lesioned area from different experimental groups. P value of less than 0.05 was presented as “*”.

Eriochrome cyanine R staining for myelin showed that in LAE animals demyelinating lesions were prominent (**Fig 4**) in regions that correspond to the areas of leukocyte infiltration on H/E. Demyelinated area was restricted to the injection site 3 days post-injection (**Fig 4**) and was more spread out into surrounding tissue 7 days after complete LAE protocol (**Fig 4**). No changes in white matter for the control groups were observed. Minor leukocyte infiltration was observed in sham-operated groups. However, there was significant difference in the level of leukocyte infiltration in injection area between the LAE and sham-operated animals.

**Fig 5:**
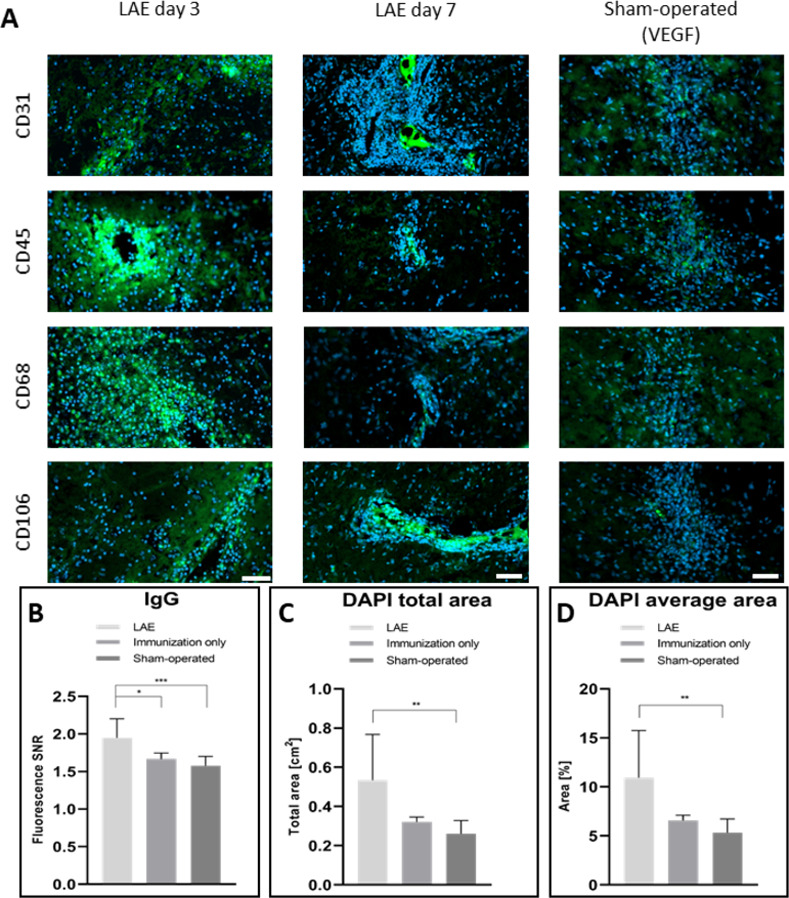
Immunofluorescent images taken from the lesioned area for various antigens (green) in injected groups, counterstained with DAPI (blue; A). Graphs display the endogenous IgG-positive fluorescent signal intensity in internal capsule with adjacent tissue, compared to control animals (B). Additionally, DAPI-specific signal was counted as a total area within the lesion (C) and as a mean percentage of total area within ROIs (D). P value of less than 0.05 was presented as “*”, <0.01 as a “**” and <0.001 as a “***”.

Using immunofluorescence, we were able to detect a positive signal for activated endothelium (CD31 and CD106 positive signal), monocytes (CD68) and leukocytes (CD45) in both 3- and 7 days post-operation (**Fig 5A**). In LAE animals, the ipsilateral hemisphere was more pronounced in IgG-specific signal (**Fig 5B**). This indicates the local BBB opening after VEGF injection (*p* < 0.001 compared to sham-operated and *p* < 0.05 compared to immunization only), pronounced, when the LAE protocol was utilized. Moreover, a robust leukocyte infiltration was detected, measured as a cell nuclei positive signal within the lesioned area (**Fig 5C and 5D**). This phenomenon is concordant with our predictions that LAE is developed in animals by local BBB opening and leukocyte infiltration.

## Discussion

Even though the EAE is the most frequently used animal model of the MS and related diseases, it is still difficult to reliably verify potential therapeutics using this technique, and therefore effective cure for is not available. Our approach to refine modelling of neurological diseases is geared towards improving the clinical relevance and creating the opportunity for local targeted therapy [11, 12]. We are aware of the enormous value of modelling autoallergic encephalopathy using EAE, but still, we hypothesize that the model itself could be modified into more replicable form. Additional problem with utilizing EAE for targeted therapy testing is the lesion placement. Histological features of EAE presents the autoimmune lesion development in whole CNS, predominantly in spinal cord and brainstem. However, when using the potential local therapy for autoimmune demyelination, EAE could be a misleading model. Thus, our LAE approach in rat brain, using immunization without any neurological impairment, followed by selective BBB disruption using VEGF is the first known model to facilitate the advantages of EAE with additional replicability and precision of the toxic demyelination models. We have also changed the target of local BBB opening, comparing to other studies, creating a lesion in brain parenchyma, instead of spinal cord tissue [13, 14]. We have decided to use a more robust immunogen- bovine spinal cord homogenate, instead of synthetic peptides. This enabled us to mount peripheral autoimmune response, with desirable lack of spontaneous brain lesions when the BBB was intact. Moreover, the less narrow immune response can be more clinically relevant as in case of MS, there is no one specific target for the autoimmune response. The bovine MBP despite species mismatch is still likely to induce EAE in rats, as presented in Hashim et al. [15]. Obviously we omitted the use of the pertussis toxin- a compound which is meant to induce activation of cerebral endothelium and a robust BBB breach. Immunization with the bovine spinal cord homogenate could potentially induced broader than intended autoimmunity including damage to axons or neurons. However, we did not observe these features in this study. Also, most of the studies and protocols suggest using rats of specific gender and strain, namely female Lewis or Dark Agouti rats, to obtain a replicable neurological deficit [5, 14, 16, 17]. In contrary, we used less susceptible Wistar rat strain to diminish the adverse effects from overreactive disease development- a problem usually connected with relatively high mortality rate in EAE [18]. Our LAE model resembles the single lesion development for more accurate potential therapy assessment with diminished death occurrence in experimental setup.

We were able to develop a subclinical, yet replicable autoimmunological response, followed by local BBB opening. The success was confirmed by the development of autoantibodies in immunized groups, and in *post-mortem* histological findings in LAE group, comparing to control animals. Moreover, as shown by the animals’ bodyweight dynamics, the immunization itself does not elicit the disease development, where in typical model, animals lose their weight significantly [19, 20]. In standard EAE disease course, the animals start to lose their weight around 1–3 days before the neurological outcome starts [19]. Also, we used a cylinder behavioral testing to assess the potential limb use asymmetry. The results are indicating the changes in post-surgery forelimb usage with an increase in contralateral forelimb usage. The data from other focal brain injury models [21, 22] suggests the otherwise- increase in ipsilateral side. However, in our approach, the neuronal necrosis is rather low, and the most abundant brain tissue pathology is an inflammatory response with demyelination. Thus, the potential compensatory behavior can be considered as an explanation for this inconsistency. Furthermore, we performed the basic characteristic of the lesion site. Histological staining using hematoxylin/eosin revealed the leukocyte infiltrates abundant in tissue samples after LAE protocol. Three days after lesion induction leukocyte infiltration was evident in the internal capsule, with demyelination in the same area as shown by eriochrome cyanine R staining (loss of myelin-specific blue color in the white matter). More widespread perivascular cuffing was observed after 7 days, but still, with prominent demyelination in the internal capsule. After both 3 and 7 days, the occurrence of characteristic perivascular cuffs was detected. This proves the autoimmune nature of the lesion, as the leukocytes are abundant in both fully treated groups. This feature is concordant with the histopathological images from active MS lesions [23]. Moreover, using eriochrome cyanine staining, we revealed the demyelinated area in injected internal capsule. The loss of myelin-specific color is typical to ipsilateral hemisphere and more pronounced after 7 days post-operative, suggesting the ongoing acute autoimmune demyelinating response in LAE animals. Also, the histopathological features suggest that lesion development is a dynamic process and take place as long as the BBB-opening effect of VEGF takes place- between 3 and 7 days. Moreover, increase in astrocyte activity and occurrence of CD45^+^ and CD68^+^ cells confirmed the local inflammatory response that is present in MS [24]. We also detected the presence of strong CD31 and CD106 positive signal within the perivascular cuffs in lesioned area. The activated endothelium is also a strong evidence of active MS lesion development. The CD31-positiveness within the lesioned area strongly suggest the autoimmunological response with leukocyte infiltration [25, 26]. Also, upregulation of CD31 is positively connected with lymphocytes diapedesis, an additional factor promoting the neuroinflammatory response within the brain tissue [27]. Another important finding is a positive reactivity against CD106, a protein which promotes the BBB permeability during neuroinflammation [28]. This target is proved to be found in animals with active EAE [29] and positive detection of the antigen was found in tissue sections in animals with LAE. Also, in MS patients, a positive staining for CD106 can be observed [30, 31]. Overall, both CD31 and CD106 are abundant in neuroinflammatory response, a phenomenon observed in our study. It is good to note that this feature is observed only in animals with LAE.

## Conclusions

All in all, our study has led us to conclude that this approach is sufficient to develop a local neuroinflammatory lesion in rat brain. The pathological hallmark is similar to that found in EAE or MS and also is highly replicable, developed in male Wistar rats. This finding is proved to be straightforward, even though the surgical procedure is required. Additionally, local character of lesion development makes the model highly valuable, especially when therapeutics are ought to be utilized.
